# Development of Metacognition in Adolescence: The Congruency-Based Metacognition Scale

**DOI:** 10.3389/fpsyg.2020.565231

**Published:** 2021-01-06

**Authors:** Kelssy Hitomi dos Santos Kawata, Yuki Ueno, Ryuichiro Hashimoto, Shinya Yoshino, Kazusa Ohta, Atsushi Nishida, Shuntaro Ando, Hironori Nakatani, Kiyoto Kasai, Shinsuke Koike

**Affiliations:** ^1^Center for Evolutionary Cognitive Sciences, Graduate School of Art and Sciences, The University of Tokyo, Tokyo, Japan; ^2^Center for Advanced School Education and Evidence-based Research (CASEER), Graduate School of Education, The University of Tokyo, Tokyo, Japan; ^3^Medical Institute of Developmental Disabilities Research, Showa University, Tokyo, Japan; ^4^Department of Language Sciences, Tokyo Metropolitan University, Tokyo, Japan; ^5^Research Center for Language, Brain and Genetics, Tokyo Metropolitan University, Tokyo, Japan; ^6^Graduate School of Letters, Arts and Sciences, Waseda University, Tokyo, Japan; ^7^Department of Neuropsychiatry, Graduate School of Medicine, The University of Tokyo, Tokyo, Japan; ^8^Department of Psychiatry and Behavioral Sciences, Tokyo Metropolitan Institute of Medical Science, Tokyo, Japan; ^9^Department of Information Media Technology, School of Information and Telecommunication Engineering, Tokai University, Tokyo, Japan; ^10^UTokyo Institute for Diversity & Adaptation of Human Mind (UTIDAHM), The University of Tokyo, Tokyo, Japan; ^11^The International Research Center for Neurointelligence (IRCN), The University of Tokyo, Tokyo, Japan; ^12^UTokyo Center for Integrative Science of Human Behaviour (CiSHuB), The University of Tokyo, Tokyo, Japan

**Keywords:** child, adolescent, metacognition, scale development, validity

## Abstract

**Introduction:**

Previous studies on metacognitive ability were explored using self-report questionnaires that are difficult to adequately measure and evaluate when the capacity for self-reference is undeveloped. This study aimed to validate the Congruency-based Metacognition Scale (CMS) to measure metacognition and the feeling of confidence abilities and to investigate the development of metacognition during adolescence.

**Methods:**

The CMS was administered to 633 child–parent pairs in Japan (child, mean age = 16.0 years, 46.0% female; parent, mean age = 48.3 years, 94.9% mother). The CMS metacognition score was assessed based on congruency scores between the self-report of the child from a third-person perspective (3PP) and the parent’s report from the first-person perspective (1PP). The CMS self-judgment accuracy score was assessed by the congruency scores between the children’s self-report from the 1PP and 3PP. For both measures, the more distant the 3PP on the self-report was from the 1PP on the parent’s report and child self-report means low ability. An exploratory factor analysis (EFA) was conducted to examine construct validity and then a confirmatory factor analysis (CFA) was used. Criterion validity was examined by calculating Pearson’s correlation coefficients with scores on the Beck Cognitive Insight Scale (BCIS) and Autism Quotient (AQ). We used intraclass correlation and Cronbach’s alpha to examine the test–retest and internal consistency reliability.

**Results:**

Based on the results of the EFA and CFA, we adopted one factor structure with five items. The CMS metacognition and CMS self-judgment accuracy showed evidence criterion validity, exhibiting significant correlations with the BCIS self-reflectiveness (*r* = 0.16) and self-certainty scores (*r* = 0.17), respectively. Regarding to the AQ, only the CMS metacognition score had significant correlations with the social skills (*r* = 0.22) and total scale score (*r* = 0.20). The test–retest reliability showed adequate (intraclass correlation coefficient 0.70–0.81 and the Cronbach’s alpha coefficient 0.63–0.59). Adolescents were found to have significantly lower metacognitive ability compared to young adults.

**Conclusion:**

CMS could be a valid and reliable measure to examine metacognitive abilities for adolescents.

## Introduction

Metacognition is commonly referred to as thinking about thinking (i.e., reflecting on one’s own thinking) (Flavell, 1979). The usage of metacognition has developed over 40 years since the first term was introduced (Flavell, 1979), for example, level of confidence in a cognitive action (monitoring), behavioral regulation (control), judgment of learning, executive skills, higher-order skills, and feeling of knowing and psychological status (Veenman et al., 2006; Fleming et al., 2010). Therefore, some reports defined metacognition as cognitive and mental processes to know and regulate their behaviors and psychological status, whereas others as the awareness and understandings of their status itself. In this study, we used metacognition as the awareness and understandings of their status (Flavell, 1979), which was defined as individual’s self-conscious supervision of the cognitive processes. To see the metacognition, we intend to use the combination of children’s third-person perspective (3PP) and parent’s first-person perspective (1PP) reports regarding children’s personal trait.

The assessment of metacognitive ability has been generally used with self-report questionnaires (Wolters et al., 2012; Lachat Shakeshaft et al., 2020). Self-report questionnaires are easy to administer and frequently used to assess metacognitive ability; however, these self-reports present several limitations, for example, it is difficult to evaluate for people with difficulties in self reference (e.g., autism spectrum disorders) and who are still developing (e.g., adolescence) (Sebastian et al., 2008). In psychology, parental reports are commonly used to assess temperament (De Los Reyes and Kazdin, 2005; Fox et al., 2005; Durbin et al., 2007; Hash et al., 2019), emotional difficulties (Theunissen et al., 2019), behavioral problems (Fujikawa et al., 2016, 2018), and depression (Park et al., 2019) in children, as they are often incapable of accurately reporting how they feel. Yet, important questions remain concerning whether the constructs assessed by the parental reports can be used to verify metacognitive ability in children.

Some researchers (Ruby and Decety, 2001; Ruby et al., 2009; Hashimoto et al., 2017; Brown et al., 2019) have combined the concept of self and the other thoughts with the 1PP and 3PP taking. Functional magnetic resonance imaging (fMRI) studies on perspective taking have observed significant activity associated with brain regions related to social cognition, including the medial prefrontal cortex (mPFC), ventromedial prefrontal cortex (vmPFC), dorsomedial prefrontal cortex (dmPFC), and posterior cingulate cortex (PCC)/precuneus. Activities in the dmPFC and PCC/precuneus have been reported during 3PP judgment, particularly when contrasted with 1PP judgment (Ruby and Decety, 2001; Ruby et al., 2009). When comparing 3P self-perspective (3PP_Self) to 1P self-perspective (1PP_Self), significant activity was observed in the PCC/precuneus (Hashimoto et al., 2017). According to a previous study, the PCC/precuneus is specifically involved in distinguishing self-produced actions from those generated by others (Ruby and Decety, 2001). In another study (Brown et al., 2019), dmPFC and vmPFC deactivation was observed while participants/actors pretended to be Romeo (male participants) or Juliet (female participants) from Shakespeare’s famous drama. These findings suggest that assessing perspective taking would more appropriately reflect metacognitive ability compared to self/child and/or other/parental questionnaires. However, to date, no study has explored whether a questionnaire on perspective taking could assess metacognitive ability in developing individuals.

This study served to develop the Congruency-based Metacognition Scale (CMS) to measure metacognitive and self-judgment accuracy abilities. The CMS metacognition score was assessed based on congruency between the child’s self-report from a third-person perspective (3PP_Self; e.g., “Does your mother/father think you are kind?”) and the parental report from the first-person perspective (1PP_Other; e.g., “Do you think your son/daughter is kind?”). The CMS self-judgment accuracy score was assessed by the congruency between the children’s self-report from a first-person perspective (1PP_Self; e.g., “Do you think you are kind?”) and third-person perspective (3PP_Self). For both measures, the more distant the 3PP on the self-report was from the 1PP on the parent’s report and child self-report means low ability. The study aimed to examine the construct, criterion-related validity, and test–retest reliability of the CMS as well as investigate metacognitive development during adolescence. Regarding the development, this study hypothesized that, consistent with normative development, children/adolescents would present with lower metacognitive ability relative to late adolescents/young adults.

## Materials and Methods

### Participants and Procedures

A total of 633 child–parent pairs participated in this study using three surveys from the Tokyo prefecture between September 2013 and January 2020 (child, mean age = 16.0 years, *SD* = 3.1, age range = 10.8–29.3 years, 46.0% female; parent, mean age = 48.3 years, *SD* = 5.0, age range = 34.0–66.0 years, 94.9% mother; see Table 1). In the first survey, 152 pairs were recruited *via* an authorized job recruitment board or internet site at 20 colleges or universities between November 2013 and July 2014 (child, mean age = 19.8 years, *SD* = 1.2, age range = 18.0–25.9 years, 42.1% female; parent, mean age = 51.5 years, *SD* = 3.6, age range = 44.0–62.0 years, 94.7% mother). In the second survey, 74 pairs were recruited from recruitment boards placed in several high schools and universities, and a commercial internet advertisement between May 2017 and October 2018 (child, mean age = 19.6 years, *SD* = 2.5, age range = 15.5–29.3 years, 51.4% female; parent, mean age = 52.7 years, *SD* = 4.8, age range = 41.0–66.0 years, 91.9% mother). In the third survey, 407 pairs were assessed in the TEEN Cohort project between September 2013 and January 2020 (child, mean age = 13.9 years, *SD* = 1.2, age range = 10.8–17.3 years, 46.4% female; parent, mean age = 46.4 years, *SD* = 4.4, age range = 34.0–58.0 years, 95.6% mother) (Okada et al., 2018; Ando et al., 2019). Following the third survey, 36 child–parent pairs were assessed on two occasions for test–retest reliability by an interval mean of 58.7 days (mean age = 16.0 years, *SD* = 0.7, age range = 15.0–16.9 years, 34.1% female; parent, mean age = 48.7 years, *SD* = 4.3, age range = 40.1–56.3 years, 91.7% mother) between July 2019 and January 2020. All participants received the questionnaires by post and responded alone at home.

**TABLE 1:** Sample characteristics and assessment scores [mean (SD)].

	Survey 1	Survey 2	Survey 3
Number of pairs	152	74	407
**Children**			
Age (years)	19.8 (1.2)	19.6 (2.5)	13.9 (1.2)
Sex (female/male)	64/88	38/36	218/189
**BCIS**			
Self-reflectiveness	15.8 (3.1)	–	–
Self-certainty	7.8 (2.8)	–	–
Composite score	8.0 (4.4)	–	–
**AQ-J-50**			
Social skills	4.4 (2.9)	–	–
Attention switching	5.2 (1.8)	–	–
Attention to detail	4.6 (2.3)	–	–
Communication	3.9 (2.2)	–	–
Imagination	4.1 (2.0)	–	–
Total	22.3 (7.2)	–	–
**Communication with parent**			
***Via* direct contact (%)**			
Almost every day	68.7	71.0	–
At least once a week	1.4	2.9	–
At least once a month	1.4	4.3	–
Less than once a month	28.6	21.7	–
***Via* telephone (%)**			
Almost every day	9.5	7.2	–
At least once a week	17.0	18.8	–
At least once a month	36.7	30.4	–
Less than once a month	36.7	43.5	–
***Via* email (%)**			
Almost every day	25.2	31.9	–
At least once a week	31.3	33.3	–
At least once a month	32.0	23.2	–
Less than once a month	11.6	11.6	–
**CMS**			
CMS metacognition	3.5 (2.1)	3.5 (2.1)	4.7 (2.6)
CMS self-judgment accuracy	2.7 (1.6)	3.2 (2.6)	2.8 (2.2)
**Parents**			
Age (years)	51.5 (3.6)	52.7 (4.8)	46.4 (4.4)
Sex (F/M/unknown)	144/7/1	68/4/2	389/17/1
**Communication with child**			
***Via* direct contact (%)**			
Almost every day	62.6	76.8	–
At least once a week	3.4	0	–
At least once a month	2.7	1.4	–
Less than once a month	31.3	21.7	–
***Via* telephone (%)**			
Almost every day	5.4	7.2	–
At least once a week	14.3	18.8	–
At least once a month	32.7	30.4	–
Less than once a month	47.6	43.5	–
***Via* email (%)**			
Almost every day	29.9	33.3	–
At least once a week	37.4	44.9	–
At least once a month	24.5	10.1	–
Less than once a month	8.2	11.6	–

*SD, standard deviation; BCIS, Beck Cognitive Insight Scale; Composite score = self-reflectiveness – self-certainty; AQ-J-50, Japanese version of 50-item Autism-Spectrum Quotient; CMS, Congruency-based metacognition scale; F, female; M, male.*

### Ethics Statement

This study was approved by the Research Ethics Committee at The University of Tokyo (No. 18-50) and the Department of Medicine at The University of Tokyo [No. 10069-(21)]. All participants, or rather their caregivers (if the participant’s age was 18 years or less), provided informed consent prior to their participation in this study in accord with the Declaration of Helsinki.

### Development of the Congruency-Based Metacognition Scale

The CMS was developed based on previous studies that have examined metacognitive ability (Ruby and Decety, 2001, 2004; D’Argembeau et al., 2007; Ruby et al., 2009; Hashimoto et al., 2017). In an fMRI study (Hashimoto et al., 2017), participants were instructed to read metacognitive sentences and decide whether they agreed with the sentence by pressing a corresponding button. The task consisted a 2 × 2 factorial design: “self” or “other” and “first-person perspective (1PP)” or “third-person perspective (3PP).” The participants were shown a sentence (in Japanese) in the form of “A think(s) that B is/are C,” where A and B represent either “YOU” (self) or “YOUR MOTHER” (Other, or “YOUR FATHER” if the parent that responded was one’s father) and C represents an adjective describing a personality trait (e.g., “YOUR MOTHER thinks that YOU are kind”). Our fMRI task used 88 Japanese adjectives translated by a native Japanese speaker (R.H.) from 110 English adjectives describing personality traits (Bochner and Van Zyl, 1985); these were also selected based on 20 Japanese volunteers, who rated how much they liked every adjective using a seven-level Likert scale. A previous study confirmed the equal characteristics in the number of letters, moras, word frequency, and imaginability among the 88 adjectives (Hashimoto et al., 2017). Of these, 20 highly familiar and frequently employed words were used in this study, with a counterbalance of 10 positive (e.g., kind, affable, brave, polite, graceful, persistent, serious, artistic, organized, and cheerful) and 10 negative words (e.g., quick-tempered, snobby, scruffy, unkind, noisy, hardheaded, talkative, boring, snaky, and irritable); see Supplementary Appendix A–F. The same set of 20 trait adjectives was used in the factor analysis of our current study.

The CMS was adapted for two domains in children (first person self: 1PP_Self and third person self: 3PP_Self) and one domain for their parents (first person other: 1PP_Other; see Table 2). The 1PP_Self condition requires a child to evaluate adjectives describing their own subjective feelings (e.g., “Do you think you are kind?”), whereas the 1PP_Other condition requires his/her parents to evaluate adjectives describing the subjective feelings of their children (e.g., “Do you think your son/daughter is kind?”). In the 3PP_Self condition, the child is asked to estimate how his/her parent would assess his/herself with respect to the adjectives (e.g., “Does your mother/father think you are kind?”). For each statement, the participants responded using a four-point Likert-type scale (1 = *disagree*, 2 = *somewhat disagree*, 3 = *somewhat agree*, and 4 = *agree*).

**TABLE 2:** Scheme of the Congruency-based Metacognition Scale (CMS) metacognition and self-judgment accuracy.

	Self-personality	Other’s personality
First-person perspective (1PP)	“Do you think you are kind?” (1PP_Self)	“Do you think your son/daughter is kind?” (1PP_Other)
Third-person perspective (3PP)	“Does your mother/father think you are kind?” (3PP_Self)	–

*Each statement is characterized with five adjectives describing a personality trait of the children, using the children’s own first-person perspective (self-personality: 1PP_Self) and third-person perspective (self-personality: 3PP_Self) and the parent’s perspective (other’s personality: 1PP_Other). For each statement, the participants responded using a four-point Likert scale (1 = disagree, 2 = somewhat disagree, 3 = somewhat agree, 4 = agree). The CMS metacognition part that measures metacognition was obtained by the sum of absolute differences between each adjective of 3PP_Self and 1PP_Other scores. The CMS self-judgment accuracy was defined as the sum of absolute differences between each adjective of 1PP_Self and 3PP_Self scores.*

We subsequently conducted a preliminary test to confirm whether 10-year-old children (*n* = 26) and their mothers (*n* = 26) were able to respond to the CMS questions without difficulty. We interviewed the participants after they completed the CMS and confirmed that all participants understood the questionnaire and responded appropriately.

The CMS metacognition variables was defined using the absolute differences between each adjective of the 3PP_Self and 1PP_Other scores. The CMS self-judgment accuracy score was also measured by CMS and defined as the absolute differences between each adjective of the 1PP_Self and 3PP_Self scores. After the construct validity discussed in the Statistical Analysis section, the sum of absolute differences between each adjective of the 3PP_Self and 1PP_Other scores and 1PP_Self and 3PP_Self scores was considered as CMS metacognition and CMS self-judgment accuracy, respectively (lower scores corresponding to greater capacity in both measurements).

### Measurement for Criterion Validity

From the first survey, we acquired children’s scores on the Beck Cognitive Insight Scale (BCIS) (Beck et al., 2004; Uchida et al., 2009) and the 50-item Autism Quotient (AQ-50) (Wakabayashi et al., 2004) for criterion validity.

#### The Beck Cognitive Insight Scale

The BCIS consists of 15 items that assess self-reflectiveness and self-certainty. Originally, the BCIS was developed to evaluate reflectiveness and overconfidence in the interpretations of the experiences of patients with mental health problems (Beck et al., 2004; Uchida et al., 2009). Items that comprise the self-reflectiveness subscale measure objectivity, reflectiveness, and openness to feedback. The self-certainty subscale assesses decision-making and dogmatic certainty regarding beliefs and conclusions. Therefore, in general, the BCIS is used as a self-report measure of the ability to reflect on personal cognitive perceptions and beliefs and to re-evaluate subjective interpretations. Thus, the BCIS would be a suitable scale for testing the validity of the CMS metacognitive and self-judgment accuracy abilities. Using the Japanese version of the BCIS (BCIS-J) (Uchida et al., 2009), respondents rated their agreement on a four-point Likert-type scale from 0 (*do not agree at all*) to 3 (*agree completely*). The self-reflectiveness subscale was calculated as the sum of nine items (possible range 0–27, with higher scores corresponding to increased self-reflectiveness). The self-certainty subscale was calculated as the sum of the remaining six items (possible range 0–18, with higher scores corresponding to more overconfidence). It was hypothesized that higher levels of self-certainty would diminish an individual’s capacity for self-reflection, so a composite score was calculated by subtracting the self-certainty total from the self-reflectiveness total, and this was used as the principal indicator of cognitive insight (Beck et al., 2004). The range of the composite score of cognitive insight is −18 through 27, with higher scores indicating better cognitive insight.

#### The Autism-Spectrum Quotient

The original AQ (Baron-Cohen et al., 2001) consists of a 50-item self-report questionnaire, designed to assess autistic spectrum traits in the general population (Skuse et al., 2005; Ronald and Hoekstra, 2011). In a general population, autism traits measured by the AQ provide evidence of a reliable association with metacognition (Carpenter et al., 2019). Thus, the AQ would be an appropriate scale for testing the validity of the CMS metacognition and self-judgment accuracy. The AQ is divided into five different areas of functioning related to autistic traits: social skills, attention switching, attention to detail, communication, and imagination. Using the Japanese version of the AQ (AQ-J-50) (Wakabayashi et al., 2004), participants rated their agreement on a four-point Likert-type scale from 1 (*definitely disagree*) to 4 (*definitely agree*). Higher scores indicated more autism-related cognitive traits. Please see the original version of the AQ (Baron-Cohen et al., 2001) for scoring.

#### The Child–Parent Communication

We inquired into the levels of child–parent communication using three questions on both the children and their parents in surveys 1 and 2. The questions were related to frequency of direct contact (“How often do you usually meet with your parents/child?”), telephone (“How often do you usually call your parents/child?”), and email (“How often do you usually send emails to your parents/child?”) communication. Participants were asked to rate their own frequency of communication using a four-point Likert scale (1: *almost every day*, 2: *at least once a week*, 3: *at least once a month*, and 4: *less than once a month*). The total score from a child response was used as the level of communication between child and parent.

### Statistical Analysis

We performed an exploratory factor analysis (EFA) to explore factor structure of the CMS metacognition and CMS self-judgment accuracy. Two EFAs (metacognition and self-judgment accuracy) were evaluated using maximum likelihood estimation with Promax rotation. Eigenvalues ≥ 1.00 were considered to indicate factors. Our approach to item adaptation was both statistical and structural. We included the items that loaded greater than 0.35 in the first and second EFAs and were found in both measures (CMS metacognition and self-judgment accuracy) in the second EFA.

After selecting the final items from the 20 items through the EFAs, a confirmatory factor analysis (CFA) was conducted for the selected items to test the fitness of the data to the one factor. We applied the following fit parameters: root mean square error of approximation (RMSEA ≤ 0.05 good fit and ≤ 0.08 acceptable fit); standardized root mean squared residual (SRMR ≤ 0.05 good fit and ≤ 0.10 acceptable fit); goodness-of-fit index (GFI), adjusted goodness-of-fit index (AGFI); and comparative fit index (CFI), with values ≥ 0.90 considered acceptable (McDonald and Ho, 2002).

The criterion validity was assessed using a Pearson’s correlation coefficient between the CMS metacognition and self-judgment accuracy scores and the BCIS and AQ-J-50 scores. A Pearson’s correlation coefficient was also performed to test the potential influence of child–parent communication on CMS scores. The strength of correlation coefficient was set as weak (*r* ≤ 0.4), moderate (0.4 < *r* ≤ 0.7), and strong (*r* > 0.7) (Dancey and Reidy, 2007).

Reliability of the scale was assessed by the intraclass correlation coefficient (ICC) and Cronbach’s alpha coefficient for the internal consistency. The ICC was conducted through the two-way mixed-effect model and absolute agreement type. An ICC of over 0.6 was considered acceptable (Weir, 2005). An alpha value above 0.7 was considered to indicate good internal consistency, and values between 0.6 and 0.7 can be accepted providing other indicators are good (Mayers, 2013).

To test the development of metacognition during adolescence measured using the CMS metacognition and self-judgment accuracy scores, we compared the linear and non-linear regression models with age as an independent variable on all participant samples. All the analyses were performed using the Statistical Package for Social Sciences (SPSS) version 25 and AMOS version 25 (IBM SPSS Inc. Chicago, IL, United States) for Windows. The level of statistical significance was set at *p* < 0.05.

## Results

### Sample Characteristics

Descriptive characteristics are shown in Table 1. A one-way ANOVA revealed a significant difference between surveys in the CMS metacognition [*F*(2, 630) = 15.4, *p* < 0.01] but not in CMS self-judgment accuracy [*F*(2, 630) = 1.4, *p* = 0.24]. For the CMS metacognition, a Bonferroni *post-hoc* test showed that survey 3 (mean = 4.7, *SD* = 2.6) presented with a significantly higher score (i.e., lower metacognitive ability) compared to surveys 1 (mean = 3.5, *SD* = 2.1) and 2 (mean = 3.5, *SD* = 2.1).

In terms of the concordance between parent and child communicative reports, the three communicative measures were observed as moderate to strong, ranging from 0.53 to 0.88 (direct contact *r* = 0.88, *via* telephone *r* = 0.58, and *via* email *r* = 0.53; *p* < 0.01). The correlations between the level of communication and the CMS scores were not significant (metacognition: *r* = 0.07, *p* = 0.33; self-judgment accuracy: *r* = 0.01, *p* = 0.86).

### Construct Validity

In the first EFA, six items for the CMS metacognition (polite, serious, kind, affable, graceful, and boring) and 10 items for the self-judgment accuracy (affable, persistent, polite, graceful, kind, cheerful, brave, serious, boring, and unkind) had greater than 0.35 factor loadings. The second EFA for the CMS metacognition and self-judgment accuracy scales revealed one factor solution (eigenvalues = 2.13 and 2.67) explaining 35.4% and 26.7% of the variance, respectively (Table 3). Any other eigenvalues explained only small amounts of additional variance. The items loading on factor 1 represented for both scales were five positive adjectives, “polite,” “serious,” “kind,” “affable,” and “graceful;” see Supplementary Appendix A–F.

**TABLE 3:** Exploratory factor analysis of CMS metacognition and CMS self-judgment accuracy (*n* = 633).

CMS metacognition	Factor 1	CMS self-judgment accuracy	Factor 1
Before deletion of items	Eigenvalue = 3.19	Before deletion of items	Eigenvalue = 3.49

**Polite**	**0.547**	Persistent	0.496
**Serious**	**0.456**	**Affable**	**0.469**
**Kind**	**0.455**	**Graceful**	**0.459**
**Affable**	**0.449**	Cheerful	0.437
**Graceful**	**0.409**	**Polite**	**0.435**
Boring	0.384	**Kind**	**0.422**
Irritable	0.347	**Serious**	**0.386**
Brave	0.332	Unkind	0.383
Cheerful	0.324	Boring	0.371
Persistent	0.314	Brave	0.371
Organized	0.313	Snaky	0.345
Unkind	0.304	Organized	0.337
Quick-tempered	0.293	Snobby	0.328
Snaky	0.287	Irritable	0.323
Artistic	0.272	Artistic	0.309
Noisy	0.267	Noisy	0.262
Snobby	0.257	Quick-tempered	0.253
Scruffy	0.202	Hardheaded	0.245
Hardheaded	0.180	Talkative	0.232
Talkative	0.176	Scruffy	0.223

After deletion of items	Eigenvalue = 2.13	After deletion of items	Eigenvalue = 2.67

**Polite**	**0.595**	**Affable**	**0.504**
**Serious**	**0.500**	Persistent	0.484
**Kind**	**0.482**	**Polite**	**0.483**
**Affable**	**0.482**	**Graceful**	**0.464**
**Graceful**	**0.461**	**Kind**	**0.464**
Boring	0.310	Cheerful	0.445
		Brave	0.383
		**Serious**	0.377
		Boring	0.341
		Unkind	0.333

*The CMS (Congruency-based Metacognition Scale) metacognition variables were defined by the absolute differences between each adjective of the third-person perspective (self-personality: 3PP_Self) and parent’s perspective (other’s personality: 1PP_Other) scores. The CMS self-judgment accuracy was defined by the absolute differences between each adjective of the children’s own first-person perspective (self-personality: 1PP_Self) and 3PP_Self scores. Bold items are the retained items.*

The CFA were found acceptable for the one-factor models. For the CMS metacognition, SRMR = 0.05, GFI = 0.97, and AGFI = 0.92 were considered as good fit, while CFI = 0.89 and RMSEA = 0.11 were slightly below and above the reference, respectively. For the CMS self-judgment accuracy, SRMR = 0.04, GFI = 0.99, AGFI = 0.96, and CFI = 0.94 were considered as good fit, while RMSEA = 0.07 was acceptable fit.

### Criterion Validity and Reliability

There was a significant positive correlation between CMS metacognition score and the BCIS self-reflectiveness (weak, *r* = 0.16, *p* = 0.047; see Table 4). There was a significant positive correlation between CMS self-judgment accuracy score and the self-certainty scores (weak, *r* = 0.17, *p* = 0.032). No correlations were observed with the composite scores. The CMS metacognition score was also associated with the AQ-J-50 social skills subscale (weak, *r* = 0.22, *p* < 0.01; see Table 5) and total score (weak, *r* = 0.20, *p* < 0.05). Yet, no correlations were observed in relation to the CMS self-judgment accuracy. No differences in the CMS scores between genders (*p* > 0.05) were observed, and the correlations between child–parent communication and CMS scores were not significant (*p* > 0.05).

**TABLE 4:** Pearson correlations of CMS metacognition and self-judgment accuracy abilities with Beck Cognitive Insight Scale scores (*n* = 152).

	CMS metacognition	CMS self-judgment accuracy
Self-reflectiveness	0.16*	0.05
Self-certainty	0.01	0.17*
Composite scores	0.11	–0.07

CMS, Congruency-based Metacognition Scale. Composite score = self-reflectiveness score - self-certainty score; *p < 0.05.

**TABLE 5:** Pearson correlations of CMS metacognition and self-judgment accuracy with AQ-J-50 scales (*n* = 148).

	CMS metacognition	CMS self-judgment accuracy
Social skill	0.22**	–0.09
Attention switching	0.09	0.01
Attention to detail	0.16	–0.05
Communication	0.11	0.01
Imagination	0.02	0.08
AQ-J-50 total	0.20*	–0.03

CMS, Congruency-based Metacognition Scale; AQ-J-50, The Japanese version of the Autism-Spectrum Quotient; *p < 0.05, **p < 0.01.

The ICC was considered acceptable for the CMS metacognition (*r* = 0.70) and for the self-judgment accuracy (*r* = 0.81), respectively. Cronbach’s alpha values were also considered acceptable for the whole 10 items (α = 0.64) and for the five items of the CMS metacognition (α = 0.63) and CMS self-judgment accuracy (α = 0.59).

### Age Effect in the CMS Metacognitive and Self-Judgment Accuracy Abilities

**FIGURE 1:**
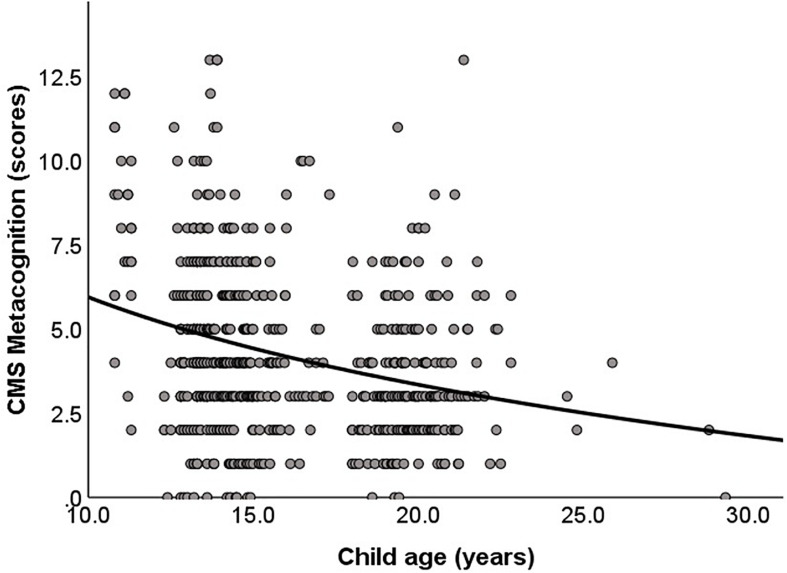
The correlation between the Congruency-based Metacognition Scale (CMS) metacognitive score and child age. The lower the CMS metacognition score, the higher the level of metacognitive ability.

A negative correlation was observed between child age and the CMS metacognition score (*r* = −0.27, *p* < 0.01), but not on self-judgment accuracy score (*r* = −0.03, *p* = 0.40). To verify the age curve-fitting of effectiveness, the regression analysis showed that both the linear model [*F*(1, 631) = 49.6, *p* < 0.01; *B* = −0.22, *B* SE = 0.03, β = −0.27, *p* < 0.01] and non-linear model (logarithmic) [*F*(1, 631) = 55.7, *p* < 0.01; *B* = −3.76, *B* SE = 0.50, β = −0.29, *p* < 0.01] were significant for CMS metacognition. On the other hand, the standard error of the regression for the non-linear model (logarithmic) was lower than the linear model (SE = 2.396 vs. 2.406), suggesting that the non-linear model (logarithmic) fit better relative to the linear model (see Figure 1). In addition, for confirmatory analysis to verify the impact that the young group and old group in the age range of 10.8–12.4 years old (*n* = 29) and 22.0–29.3 (*n* = 14) had on the current result, we excluded these age ranges and reanalyzed the correlation between child age and the CMS metacognition score. The result confirms our previous results using all participants of the negative correlation between child age and the CMS metacognition score (*r* = −0.19, *p* < 0.01).

## Discussion

This is the first study that examined the development of metacognitive ability based on child–parent response congruency in a large sample dataset from childhood to young adulthood. The present study demonstrated evidence of validity through the correlation of the CMS with the BCIS and test–retest reliability. Furthermore, CMS metacognition was positively correlated with the child AQ-J-50 total and social skills subscales, suggesting the CMS reflects the underdevelopment of metacognitive ability. Finally, a non-linear model showed that the metacognitive ability measured using CMS decreased during early adolescence.

### Criterion Validity and Reliability

CMS metacognition and self-judgment accuracy scores showed a weak correlation with BCIS self-reflectiveness and self-certainty, respectively. The weak correlation is arguably explained by the fact that CMS metacognition and self-judgment accuracy are based on using a congruency measurement and the parental report was used as a reference to measure “performance,” while the BCIS is a self-report measure. The current result is consistent with the previous findings (Kanie et al., 2014) of a study that investigated the relationship between BCIS scores and the Social Cognition Screening Questionnaire (SCSQ) metacognition subscale, a measure based on confidence levels. Moreover, in the same study (Kanie et al., 2014), it was reported that impaired metacognition involves a decreased ability to evaluate the accuracy of one’s own judgments, often due to overconfidence.

Positive correlations were found between CMS metacognition score and the AQ-J-50 total and social skills scores of the same construct, but not with CMS self-judgment accuracy. A negative correlation trend has been previously observed between the AQ score and the left PCC/precuneus as a function of adopting a 3PP_Self (Hashimoto et al., 2017). We speculate that the combination of different perspectives in the self and in the other are necessary to negatively correlate the AQ with brain structures in the CMS, such as the PCC/precuneus.

The reliability of the CMS was determined using the ICC and Cronbach’s alpha coefficient that showed acceptable test–retest reliability. It is worth noting this because numerous metacognition studies have not examined test–retest reliability (Bacow et al., 2009; Larøi et al., 2009; Hsu, 2010; Cook et al., 2014; Martin et al., 2014; Bailey and Wells, 2015; Fernie et al., 2015; Kollmann et al., 2016; Kolubinski et al., 2017; Alma et al., 2018; Caselli et al., 2018; Lloyd et al., 2018). In addition, the present findings are consistent with some previous studies (Cartwright-Hatton et al., 2004; Wilson et al., 2011; Lachat Shakeshaft et al., 2020) with respect to the stability of metacognition over the period of time, although the correlation was weak (*r* = 0.24–0.34) (Cartwright-Hatton et al., 2004) or unstable for some domains (*r* = 0.24–0.90) (Wilson et al., 2011) of the metacognitive questionnaire when comparing with our test–retest (*r* = 0.70–0.81). Regarding to the Cronbach’s alpha coefficient, although the results revealed acceptable internal reliability values for both the whole 10 items (α = 0.64) and for the five items of the CMS metacognition (α = 0.63), the exception was the CMS self-judgment accuracy for which Cronbach’s α was 0.59. This might be related to the fact that during EFA, the items of the CMS self-judgment accuracy had to be adapted to the items of the CMS metacognition. Although, if we used another way of interpreting the alpha statistic, this value could be considered as satisfactory (Taber, 2018).

### Age Effect in the CMS Metacognitive Ability

In our study, the developmental trajectory of metacognition appeared as follows: low metacognitive ability (high scores) in childhood, followed by a gradual increase metacognitive ability (low score) during adolescence to late adolescence/young adulthood. Previous studies have demonstrated that metacognitive ability tends to be higher in adolescents compared to children (Ormond et al., 1991; Flavell et al., 1998) but not late adolescents/young adults (Vukman, 2005; Weil et al., 2013). This is because during adolescence, increases in white matter volume and decreases in gray matter volume in the frontal cortex accompany aging (Dumontheil, 2014), ultimately impacting their cognitive capacity for abstraction and self-reflection (Sebastian et al., 2008).

Some previous studies on adolescents using self-report questionnaires have not found age-related increases in metacognitive beliefs (Cartwright-Hatton et al., 2004; Ellis and Hudson, 2011; Wilson et al., 2011). This discrepancy between results can be arguably explained by the methodology used to measure metacognition, as it was based on self-report measures and a limited age range (11–17 years old). Our study based our operationalization of metacognition on both the child’s (third person) and parent’s (first-person) perspectives. According to previous studies (Desoete, 2008; Saraç and Karakelle, 2017), children’s assessments (self) of their personal metacognition abilities were consistent with their teacher’s (other) assessments at the age of approximately 8–10 years old, for example. These findings suggest that metacognitive processes are complex and require assessment using a variety of perspectives. In terms of the limited age range, it is worth noting that a study has shown that 13-year-old adolescents had the highest scores on the metacognitive ability relative to older adolescents (age range: 13–17 years old), suggesting that metacognitive ability is nearly fully formed by the age of 13 years old (Cartwright-Hatton et al., 2004). Other studies found non-correlation with age, in the similar age ranges of 11–16 years old (Wilson et al., 2011) and 12–17 years old (Ellis and Hudson, 2011). On the other hand, using the same metacognition questionnaire, a significant negative correlation (age range of 13–16 years old) (Matthews et al., 2007) and a positive correlation (age range of 12–18 years old; age range of 13–17 years old) (Wolters et al., 2012; Lachat Shakeshaft et al., 2020) were found. As such, these findings further demonstrate the importance of examining metacognitive ability across a broader age range. Therefore, it is possible to conclude that, with age, the metacognitive ability improves through, in our case, the difference of the children–parental responses that become more focused and accurate—and this trajectory tend to improve in young adulthood.

The items for the scale consist of five positive adjectives. The results replicated a previous study showing that the association between parental and adolescents’ metacognitive beliefs of rumination was significant in the case of positive metacognitive belief, but not negative (Chow and Lo, 2017). The child’s response for negative personality trait could be biased especially in adolescence, and therefore, the gap with parental response may not be appropriate for the measurement of metacognition.

Three limitations were present in the current study. First, the majority of “other/parents” were represented by maternal rather than paternal figures. A future study could incorporate “other/parental” people, involved in more distant relationships with the child to represent 1PP_other in CMS metacognition. Second, survey 3 consisted of majority of the sample. Therefore, though the whole sample consisted of 633 child–parent pairs, the criterion validity with BCIS was indeed based on the sample of survey 1, which was 152 child–parent pairs. This, however, does not diminish the importance of the current validity results from the CMS metacognition values only used on the basis of survey 1. In addition, due to the fact that collecting data from children and parents simultaneously can be challenging and based on the sample size from previous studies (sample size = 42–214) (Cartwright-Hatton et al., 2004; Matthews et al., 2007; Ellis and Hudson, 2011; Wilson et al., 2011; Wolters et al., 2012; Lachat Shakeshaft et al., 2020) that investigate the validation of metacognition in adolescents, our sample size is representative enough to assess the validity of CMS metacognition. Thus, in future studies, it will be important to replicate the present study. Third, because the sample size of the children over age 22 was small, we are unable fully to generalize the method of metacognitive measurement after adolescence. One reason for smaller sample size in this study was the difficulties in recruitment for the pairs in which the children were over age 22. Therefore, the questionnaire may be more reliable for measuring for adolescents.

In conclusion, the present study demonstrates that the CMS could be a useful tool for assessing metacognition developmentally—from childhood to young adulthood. This study demonstrated that metacognitive ability appears to develop particularly in the early phases of adolescence. Although our results indicate that CMS metacognition reflects underdevelopment in metacognitive ability, in future studies, we aim to verify the utility of CMS in the assessment of autism spectrum disorders in children.

## Data Availability Statement

The datasets generated for this study are available on request to the corresponding author. The datasets for this article are not publicly available due to state restrictions, such as privacy or ethical restrictions. Requests to access the datasets should be directed to Shinsuke Koike, c-koike@g.ecc.u-tokyo.ac.jp.

## Ethics Statement

The studies involving human participants were reviewed and approved by the Research Ethics Committee at The University of Tokyo (No. 18-50) and Department of Medicine of The University of Tokyo (No. 10069-21). Written informed consent to participate in this study was provided by the participants’ legal guardian/next of kin.

## Author Contributions

KHdSK and SK conceived the experiment and drafted the manuscript. KHdSK, YU, SY, and KO collected and analyzed data. RH, AN, SA, HN, KK, and SK critically reviewed and approved the final version of the manuscript. All authors contributed to the article and approved the submitted version.

## Conflict of Interest

The authors declare that the research was conducted in the absence of any commercial or financial relationships that could be construed as a potential conflict of interest.
