# Supplementary material for: Development of Metacognition in Adolescence: The Congruency-Based Metacognition Scale
Source: Front Psychol. 2021 Jan 6;11:565231. doi: 10.3389/fpsyg.2020.565231 (PMC7815698; doi:10.3389/fpsyg.2020.565231)
Supplement: Supplementary file 1 [file Data_Sheet_1.docx]

Appendix A: The Congruency-based Metacognition Scale (CMS) - 3P_Child/Self

Japanese version

今回の研究アンケートで、養育者用のアンケートに答えたのは、あなたからみて、

どなたにあたりますか（この部分はお母さんやお父さんに聞いても大丈夫です）

□ 母 □ 父 □ その他 ( )

| 次に、**あなたのお母さん**が、あなたのことを  どう思っているか、教えてください。  （今回のアンケートに答えてくれた人がお父さんか、  その他の方であれば、その人について、答えて下さい。）  あてはまるものに✔をつけてください。 | | | そう思わない | どちらかといえば そう思わない | どちらかといえば そう思う | そう思う |
| --- | --- | --- | --- | --- | --- | --- |
| 3P_C01 | お母さんはあなたのことを、だらしない、と思っている？ | □ | □ | □ | □ |  |
| 3P_C02 | お母さんはあなたのことを、おしゃべりだ、と思っている？ | □ | □ | □ | □ |  |
| **3P_C03** | **お母さんはあなたのことを、すなおだ、と思っている？** | □ | □ | □ | □ |  |
| 3P_C04 | お母さんはあなたのことを、短気だ、と思っている？ | □ | □ | □ | □ |  |
| 3P_C05 | お母さんはあなたのことを、さわがしい、と思っている？ | □ | □ | □ | □ |  |
| **3P_C06** | **お母さんはあなたのことを、親切だ、と思っている？** | □ | □ | □ | □ |  |
| 3P_C07 | お母さんはあなたのことを、いばっている、と思っている？ | □ | □ | □ | □ |  |
| 3P_C08 | お母さんはあなたのことを、がんこだ、と思っている？ | □ | □ | □ | □ |  |
| 3P_C09 | お母さんはあなたのことを、ずるがしこい、と思っている？ | □ | □ | □ | □ |  |
| 3P_C10 | お母さんはあなたのことを、怒りっぽい、と思っている？ | □ | □ | □ | □ |  |
| 3P_C11 | お母さんはあなたのことを、きちんとしている、と思っている？ | □ | □ | □ | □ |  |
| 3P_C12 | お母さんはあなたのことを、芸術的だ、と思っている？ | □ | □ | □ | □ |  |
| 3P_C13 | お母さんはあなたのことを、いじわるだ、と思っている？ | □ | □ | □ | □ |  |
| 3P_C14 | お母さんはあなたのことを、だ、と思っている？ | □ | □ | □ | □ |  |
| **3P_C15** | **お母さんはあなたのことを、まじめだ、と思っている？** | □ | □ | □ | □ |  |
| **3P_C16** | **お母さんはあなたのことを、正直だ、と思っている？** | □ | □ | □ | □ |  |
| 3P_C17 | お母さんはあなたのことを、陽気だ、と思っている？ | □ | □ | □ | □ |  |
| 3P_C18 | お母さんはあなたのことを、つまらない、と思っている？ | □ | □ | □ | □ |  |
| 3P_C19 | お母さんはあなたのことを、ねばり強い、と思っている？ | □ | □ | □ | □ |  |
| **3P_C20** | **お母さんはあなたのことを、やさしい、と思っている？** | □ | □ | □ | □ |  |

*Note.* Bold items are the final 5 retained items

Appendix B: The Congruency-based Metacognition Scale (CMS) - 1P_ Child/Self

Japanese version

| あなたが、**自分のこと**をどう思っているか、教えてください。  あてはまるものに✔をつけてください。 | | そう思わない | どちらかといえば そう思わない | どちらかといえば そう思う | そう思う |
| --- | --- | --- | --- | --- | --- |
| 1P_C01 | あなたは自分のことを、短気だ、と思っている？ | □ | □ | □ | □ |
| **1P_C02** | **あなたは自分のことを、親切だ、と思っている？** | □ | □ | □ | □ |
| 1P_C03 | あなたは自分のことを、いばっている、と思っている？ | □ | □ | □ | □ |
| **1P_C04** | **あなたは自分のことを、やさしい、と思っている？** | □ | □ | □ | □ |
| 1P_C05 | あなたは自分のことを、だらしない、と思っている？ | □ | □ | □ | □ |
| 1P_C06 | あなたは自分のことを、だ、と思っている？ | □ | □ | □ | □ |
| 1P_C07 | あなたは自分のことを、いじわるだ、と思っている？ | □ | □ | □ | □ |
| **1P_C08** | **あなたは自分のことを、正直だ、と思っている？** | □ | □ | □ | □ |
| 1P_C09 | あなたは自分のことを、さわがしい、と思っている？ | □ | □ | □ | □ |
| **1P_C10** | **あなたは自分のことを、すなおだ、と思っている？** | □ | □ | □ | □ |
| 1P_C11 | あなたは自分のことを、がんこだ、と思っている？ | □ | □ | □ | □ |
| 1P_C12 | あなたは自分のことを、ねばり強い、と思っている？ | □ | □ | □ | □ |
| 1P_C13 | あなたは自分のことを、おしゃべりだ、と思っている？ | □ | □ | □ | □ |
| **1P_C14** | **あなたは自分のことを、まじめだ、と思っている？** | □ | □ | □ | □ |
| 1P_C15 | あなたは自分のことを、芸術的だ、と思っている？ | □ | □ | □ | □ |
| 1P_C16 | あなたは自分のことを、つまらない、と思っている？ | □ | □ | □ | □ |
| 1P_C17 | あなたは自分のことを、きちんとしている、と思っている？ | □ | □ | □ | □ |
| 1P_C18 | あなたは自分のことを、陽気だ、と思っている？ | □ | □ | □ | □ |
| 1P_C19 | あなたは自分のことを、ずるがしこい、と思っている？ | □ | □ | □ | □ |
| 1P_C20 | あなたは自分のことを、怒りっぽい、と思っている？ | □ | □ | □ | □ |

*Note.* Bold items are the final 5 retained items

Appendix C: The Congruency-based Metacognition Scale (CMS) - 1P_Parent/Other

| **お子さんについて**、どう思っているか、教えてください。  あてはまるものに✔をつけてください。 | | そう思わない | どちらかといえば そう思わない | どちらかといえば そう思う | そう思う |  |
| --- | --- | --- | --- | --- | --- | --- |
| 1P_P01 | | あなたはお子さんのことを、短気だ、と思っている？ | □ | □ | □ | □ |
| **1P_P02** | | **あなたはお子さんのことを、親切だ、と思っている？** | □ | □ | □ | □ |
| 1P_P03 | | あなたはお子さんのことを、いばっている、と思っている？ | □ | □ | □ | □ |
| **1P_P04** | | **あなたはお子さんのことを、やさしい、と思っている？** | □ | □ | □ | □ |
| 1P_P05 | | あなたはお子さんのことを、だらしない、と思っている？ | □ | □ | □ | □ |
| 1P_P06 | | あなたはお子さんのことを、勇敢だ、と思っている？ | □ | □ | □ | □ |
| 1P_P07 | | あなたはお子さんのことを、いじわるだ、と思っている？ | □ | □ | □ | □ |
| **1P_P08** | | **あなたはお子さんのことを、正直だ、と思っている？** | □ | □ | □ | □ |
| 1P_P09 | | あなたはお子さんのことを、さわがしい、と思っている？ | □ | □ | □ | □ |
| **1P_P10** | | **あなたはお子さんのことを、すなおだ、と思っている？** | □ | □ | □ | □ |
| 1P_P11 | | あなたはお子さんのことを、がんこだ、と思っている？ | □ | □ | □ | □ |
| 1P_P12 | | あなたはお子さんのことを、ねばり強い、と思っている？ | □ | □ | □ | □ |
| 1P_P13 | | あなたはお子さんのことを、おしゃべりだ、と思っている？ | □ | □ | □ | □ |
| **1P_P14** | | **あなたはお子さんのことを、まじめだ、と思っている？** | □ | □ | □ | □ |
| 1P_P15 | | あなたはお子さんのことを、芸術的だ、と思っている？ | □ | □ | □ | □ |
| 1P_P16 | | あなたはお子さんのことを、つまらない、と思っている？ | □ | □ | □ | □ |
| 1P_P17 | | あなたはお子さんのことを、きちんとしている、と思っている？ | □ | □ | □ | □ |
| 1P_P18 | | あなたはお子さんのことを、陽気だ、と思っている？ | □ | □ | □ | □ |
| 1P_P19 | | あなたはお子さんのことを、ずるがしこい、と思っている？ | □ | □ | □ | □ |
| 1P_P20 | | あなたはお子さんのことを、怒りっぽい、と思っている？ | □ | □ | □ | □ |

Japanese version

*Note.* Bold items are the final 5 retained items

Appendix D: The Congruency-based Metacognition Scale (CMS) - 3P_Child/Self

English version

What kind of relationship do you have with the person who answered the parent's questionnaire? (You can ask this part to your mother or your father)

□ Mother □ Father □ Other ( )

| Please tell me what does your **mother** think of you?  (If the person who answered this questionnaire is father or others,  please answer about that person.)  Please ✔(check) those that apply. | | | Disagree | Somewhat disagree | Somewhat agree | Agree |
| --- | --- | --- | --- | --- | --- | --- |
| 3P_C01 | Does your mother think you are scruffy? | □ | □ | □ | □ |  |
| 3P_C02 | Does your mother think you are talkative? | □ | □ | □ | □ |  |
| **3P_C03** | **Does your mother think you are graceful?** | □ | □ | □ | □ |  |
| 3P_C04 | Does your mother think you are quick-tempered? | □ | □ | □ | □ |  |
| 3P_C05 | Does your mother think you are noisy? | □ | □ | □ | □ |  |
| **3P_C06** | **Does your mother think you are kind?** | □ | □ | □ | □ |  |
| 3P_C07 | Does your mother think you are snobby? | □ | □ | □ | □ |  |
| 3P_C08 | Does your mother think you are hardheaded? | □ | □ | □ | □ |  |
| 3P_C09 | Does your mother think you are snaky? | □ | □ | □ | □ |  |
| 3P_C10 | Does your mother think you are irritable? | □ | □ | □ | □ |  |
| 3P_C11 | Does your mother think you are organized? | □ | □ | □ | □ |  |
| 3P_C12 | Does your mother think you are artistic? | □ | □ | □ | □ |  |
| 3P_C13 | Does your mother think you are unkind? | □ | □ | □ | □ |  |
| 3P_C14 | Does your mother think you are brave? | □ | □ | □ | □ |  |
| **3P_C15** | **Does your mother think you are serious?** | □ | □ | □ | □ |  |
| **3P_C16** | **Does your mother think you are polite?** | □ | □ | □ | □ |  |
| 3P_C17 | Does your mother think you are cheerful? | □ | □ | □ | □ |  |
| 3P_C18 | Does your mother think you are boring? | □ | □ | □ | □ |  |
| 3P_C19 | Does your mother think you are persistent? | □ | □ | □ | □ |  |
| **3P_C20** | **Does your mother think you are affable?** | □ | □ | □ | □ |  |

*Note.* Bold items are the final 5 retained items

Appendix E: The Congruency-based Metacognition Scale (CMS) - 1P_ Child/Self

English version

| Please tell me what do you think of **yourself**?  Please ✔(check) those that apply. | | Disagree | Somewhat disagree | Somewhat agree | Agree |
| --- | --- | --- | --- | --- | --- |
| 1P_C01 | Do you think you are quick-tempered? | □ | □ | □ | □ |
| **1P_C02** | **Do you think you are kind?** | □ | □ | □ | □ |
| 1P_C03 | Do you think you are snobby? | □ | □ | □ | □ |
| **1P_C04** | **Do you think you are affable?** | □ | □ | □ | □ |
| 1P_C05 | Do you think you are scruffy? | □ | □ | □ | □ |
| 1P_C06 | Do you think you are brave? | □ | □ | □ | □ |
| 1P_C07 | Do you think you are unkind? | □ | □ | □ | □ |
| **1P_C08** | **Do you think you are polite?** | □ | □ | □ | □ |
| 1P_C09 | Do you think you are noisy? | □ | □ | □ | □ |
| **1P_C10** | **Do you think you are graceful?** | □ | □ | □ | □ |
| 1P_C11 | Do you think you are hardheaded? | □ | □ | □ | □ |
| 1P_C12 | Do you think you are persistent? | □ | □ | □ | □ |
| 1P_C13 | Do you think you are talkative? | □ | □ | □ | □ |
| **1P_C14** | **Do you think you are serious?** | □ | □ | □ | □ |
| 1P_C15 | Do you think you are artistic? | □ | □ | □ | □ |
| 1P_C16 | Do you think you are boring? | □ | □ | □ | □ |
| 1P_C17 | Do you think you are organized? | □ | □ | □ | □ |
| 1P_C18 | Do you think you are cheerful? | □ | □ | □ | □ |
| 1P_C19 | Do you think you are snaky? | □ | □ | □ | □ |
| 1P_C20 | Do you think you are irritable? | □ | □ | □ | □ |

*Note.* Bold items are the final 5 retained items

Appendix F: The Congruency-based Metacognition Scale (CMS) - 1P_Parent/Other

English version

| Please tell me what do you think of your child?  Please ✔(check) those that apply. | | Disagree | Somewhat disagree | Somewhat agree | Agree |  |
| --- | --- | --- | --- | --- | --- | --- |
| 1P_P01 | | Do you think your son/daughter is quick-tempered? | □ | □ | □ | □ |
| **1P_P02** | | **Do you think your son/daughter is kind?** | □ | □ | □ | □ |
| 1P_P03 | | Do you think your son/daughter is snobby? | □ | □ | □ | □ |
| **1P_P04** | | **Do you think your son/daughter is affable?** | □ | □ | □ | □ |
| 1P_P05 | | Do you think your son/daughter is scruffy? | □ | □ | □ | □ |
| 1P_P06 | | Do you think your son/daughter is brave? | □ | □ | □ | □ |
| 1P_P07 | | Do you think your son/daughter is unkind? | □ | □ | □ | □ |
| **1P_P08** | | **Do you think your son/daughter is polite?** | □ | □ | □ | □ |
| 1P_P09 | | Do you think your son/daughter is noisy? | □ | □ | □ | □ |
| **1P_P10** | | **Do you think your son/daughter is graceful?** | □ | □ | □ | □ |
| 1P_P11 | | Do you think your son/daughter is hardheaded? | □ | □ | □ | □ |
| 1P_P12 | | Do you think your son/daughter is persistent? | □ | □ | □ | □ |
| 1P_P13 | | Do you think your son/daughter is talkative? | □ | □ | □ | □ |
| **1P_P14** | | **Do you think your son/daughter is serious?** | □ | □ | □ | □ |
| 1P_P15 | | Do you think your son/daughter is artistic? | □ | □ | □ | □ |
| 1P_P16 | | Do you think your son/daughter is boring? | □ | □ | □ | □ |
| 1P_P17 | | Do you think your son/daughter is organized? | □ | □ | □ | □ |
| 1P_P18 | | Do you think your son/daughter is cheerful? | □ | □ | □ | □ |
| 1P_P19 | | Do you think your son/daughter is snaky? | □ | □ | □ | □ |
| 1P_P20 | | Do you think your son/daughter is irritable? | □ | □ | □ | □ |

*Note.* Bold items are the final 5 retained items
